# Vitamin D Status and Supplementation Practices in Elite Irish Athletes: An Update from 2010/2011

**DOI:** 10.3390/nu8080485

**Published:** 2016-08-09

**Authors:** Joshua Todd, Sharon Madigan, Kirsty Pourshahidi, Emeir McSorley, Eamon Laird, Martin Healy, Pamela Magee

**Affiliations:** 1 Northern Ireland Centre for Food and Health, University of Ulster, Coleraine, Londonderry BT52 1SA, Northern Ireland, UK; todd-j10@email.ulster.ac.uk (J.T.); k.pourshahidi@ulster.ac.uk (K.P.); em.mcsorley@ulster.ac.uk (E.M.); 2 Irish Institute of Sport, Sports Campus Ireland, Abbotstown, Dublin 15, Republic of Ireland; smadigan@instituteofsport.ie; 3 School of Biochemistry and Immunology, Trinity College Dublin, Dublin 2, Republic of Ireland; lairdea@tcd.ie; 4 Department of Biochemistry, Central Pathology Laboratory, St. James’s Hospital, Dublin 8, Republic of Ireland; mhealy@stjames.ie

**Keywords:** Elite athletes, vitamin D, supplementation

## Abstract

Vitamin D deficiency is a global health concern that is prevalent in Ireland. The vitamin D status of elite Irish athletes following implementation of a revised supplementation policy in 2010/2011 has not been explored to date. This study aimed to assess the vitamin D status of elite Irish athletes participating in high-profile sports and establish if equatorial travel, supplementation and/or sunbed use predict vitamin D status. Across Ireland, blood samples (*n* = 92) were obtained from cricketers (*n* = 28), boxers (*n* = 21) and women’s rugby sevens players (*n* = 43) between November 2013 and April 2015. Total 25-hydroxyvitamin D (25(OH)D) concentrations were quantified using LC-MS/MS. Parathyroid hormone and adjusted calcium concentrations were measured by clinical biochemistry. Athletes completed a questionnaire that queried equatorial travel, supplementation and sunbed use. Vitamin D sufficiency (25(OH)D >50 nmol/L) was evident in 86% of athletes. Insufficiency (31–49 nmol/L) and deficiency (<30 nmol/L) was present in only 12% and 2% of athletes respectively. On average, athletes from all sport disciplines were vitamin D sufficient and 25% reported vitamin D supplementation which was a significant positive predictor of vitamin D status, (OR 4.31; 95% CI 1.18–15.75; *p* = 0.027). Equatorial travel and sun bed use were reported in 47% and 16% of athletes respectively however these factors did not predict vitamin D status (both *p* > 0.05). Although different cohorts were assessed, the overall prevalence of vitamin D insufficiency/deficiency was 55% in 2010/2011 compared with only 14% in 2013/2015. Targeted supplementation is highly effective in optimising vitamin D status, negating the need for blanket-supplementation in elite cohorts.

## 1. Introduction

In Ireland, vitamin D insufficiency and deficiency, defined by the U.S Institute of Medicine as a total 25-hydroxyvitamin D (25(OH)D) concentration below 50 nmol/L and 30 nmol/L respectively [1], is pervasive in athletes, thus raising concern of potential skeletal and extra-skeletal health implications [2]. Chronic vitamin D deficiency (25(OH)D <30 nmol/L) clinically manifests as rickets in children and osteomalacia in adults, debilitating conditions characterised by a bow-legged posture and an increased risk of fracture [3]. Emerging research also suggests that vitamin D may be important in maintaining a healthy immune system, particularly with respect to upper respiratory tract infections which are commonly reported in athletes [4,5]. It has been suggested that a total 25(OH)D concentration of >75 or even >120 nmol/L may be considered optimal for achieving the proposed extra-skeletal health benefits of vitamin D [6,7] however there is currently insufficient evidence from randomised controlled trials to support such thresholds for athletes.

The primary source of vitamin D is cutaneous synthesis driven by ultraviolet-B radiation, at a wavelength of 290–315 nm, interacting with 7-dehydrocholesterol and forming pre-vitamin D in cells of the upper epidermis [8]. Due to Ireland’s northerly latitude (51°N–55°N), sunlight is only of sufficient strength to trigger cutaneous vitamin D synthesis for 6 months of the year (April to September); resulting in seasonal fluctuation in vitamin D status [9]. As such, geographical location is the major factor contributing to poor vitamin D status in Irish athletes as well as the general population [10,11]. In addition, indoor, early-morning and late-evening training sessions may also contribute to the prevalence of vitamin D insufficiency/deficiency in athletes [12]. Compounding this issue is the limited availability and consumption of vitamin D-rich foods by Irish athletes such as fatty fish and liver [10,13].

Previous studies conducted by our research group have identified a particularly high prevalence of vitamin D insufficiency/deficiency in Gaelic footballers, Paralympians and boxers competing at both the collegiate and elite-level [10,13]. Based on these findings the Irish Institute of Sport revised their vitamin D supplementation policy; aiming to ensure that identified cases of vitamin D insufficiency/deficiency were dealt with appropriately. It is not known, however, if the extent of this health concern extends to elite athletes competing in other high-profile sports in Ireland such as those within international cricket and rugby teams. This study therefore aimed to assess the vitamin D status of elite Irish athletes participating in a range of high-profile sports and establish if equatorial travel, supplement use and/or sunbed use are predictors of vitamin D status.

## 2. Materials and Methods

### 2.1. Recruitment

This observational study took place between November 2013 and April 2015 at training locations across the island of Ireland. The study was approved by the University Research Ethics Committee (REC/13/0235) and conducted in accordance with the declaration of Helsinki. A total of 64 elite athletes, that were actively competing internationally, were recruited through the teams’ performance dietitian and provided with an information sheet detailing the study procedures prior to obtaining informed consent. Overall, 92 blood samples were obtained from male cricketers (*n* = 28), male and female boxers (*n* = 18 and *n* = 3 respectively), and female rugby seven players (*n* = 43) across multiple time points. Samples were obtained in the months of February (cricket *n* = 14); March (rugby *n* = 7); April (boxing *n* = 18); May (cricket *n* = 15); September (cricket *n* = 13) and November (rugby *n* = 22 and boxing *n* = 3).

### 2.2. Blood Collection and Processing

Trained phlebotomists obtained blood samples from the antecubital fossa; using a 21-gauge butterfly needle and 8 mL serum and 9 mL ethylenediaminetetraacetic (EDTA) plasma tubes (Greiner Bio-One GmbH, Kremsmünster, Austria). Following inversion, serum tubes were left at room temperature for up to 60 min and EDTA plasma tubes placed in refrigeration or on ice until processing. Within 3 h of collection, tubes were centrifuged at 2200 rpm for 15 min at 4 °C. Following separation, serum and plasma samples were pipetted into 0.5 mL aliquots and stored at −80 °C until further analysis.

### 2.3. Blood Analyses

All analyses were run in duplicate. Liquid chromatography-tandem mass spectrometry (LC-MS/MS) (API 4000; AB SCIEX) was used to quantify serum 25(OH)D_2_ and 25(OH)D_3_ concentrations, using a commercially available assay (Chromsystems Instruments and Chemicals GmbH; MassChrom 25-OH-Vitamin D3/D2). This analysis was undertaken at the biochemistry department of St James’ Hospital Dublin; a laboratory that complies with the Vitamin D External Quality Assessment Scheme and use of the National Institute of Standards and Technology 972 vitamin D standard reference material. The respective inter- and intra-assay coefficients of variation were 6.5% and 7.5%. Serum calcium concentrations (adjusted for serum albumin) were determined using an ILab 650 clinical biochemistry analyser at the University of Ulster, Coleraine. Plasma parathyroid hormone (PTH) concentration was quantified at Altnagelvin area hospital using a Cobas 4000 clinical biochemistry analyser (Roche Diagnostics Ltd., Burgess Hill, UK).

### 2.4. Lifestyle Questionnaire

In the presence of a researcher, a self-reported lifestyle questionnaire was completed by each athlete in order to estimate use of dietary supplements containing vitamin D, sunbed use and equatorial travel in the 6 months prior to sampling.

### 2.5. Statistical Analysis

An a priori power calculation with significance set at *p* < 0.05 and statistical power at 95% determined that 27 athletes were required in order to detect a 31.4 nmol/L difference in total 25(OH)D concentration between sport disciplines (GPower Version 3.1) [10]. The Statistical Package for the Social Sciences (SPSS) was used for all further analyses (IBM SPSS Statistics for Windows, Version 21.0, IBM Corp., Armonk, NY, USA). Data distribution was assessed using the Shapiro-Wilk test. All measures had a skewed data distribution and were therefore log-transformed prior to hypothesis testing. For continuous variables, differences in outcome measures between sport disciplines were identified using analysis of variance (ANOVA) with Bonferroni post-hoc test. *p*-values were adjusted using the Bonferroni correction for multiple comparisons. A Chi square test was used to identify if vitamin D status varied according to season of sampling (Spring/Summer (March–August) versus Autumn/Winter (September–February)). A Chi squared test was also used to determine seasonal variation in sampling according to sex. Differences in questionnaire responses, between sport disciplines, were determined using a Chi-square test with *post-hoc* analysis comparing standardised residuals [14]. A logistic regression model was used to identify if vitamin D supplementation, sunbed use or travel to an equatorial location were significant predictors of vitamin D status. As only 2 athletes exhibited vitamin D deficiency (total 25(OH)D concentration <30 nmol/L); vitamin D status was defined as sufficiency (>50 nmol/L) and insufficiency/deficiency (<50 nmol/L).

## 3. Results

Physical and biochemical characteristics of elite athletes are detailed in Table 1.

**Figure 1:**
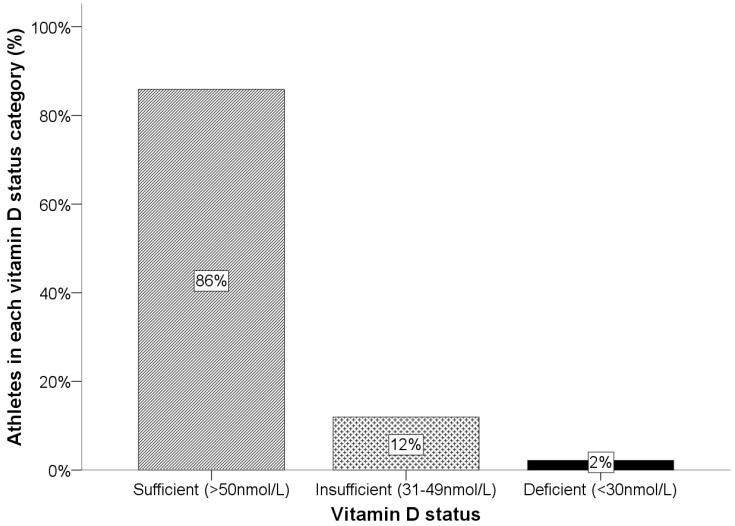
Vitamin D status of athletes. Overall, 86% of athletes were vitamin D sufficient; 12% of athletes were vitamin D insufficient (31–49 nmol/L) and 2% vitamin D deficient (<30 nmol/L).

As expected, there was a significant difference in the distribution of male and female athletes recruited from each sport discipline (rugby *n* = 43 females; boxing *n* = 18 males and *n* = 3 females; cricket *n* = 28 males) *p* < 0.001. The vitamin D status of athletes is outlined in Figure 1 and this did not vary according to season of sampling, *p* = 0.548.

Rugby players had a significantly lower mean total 25(OH)D concentration compared to boxers and cricketers. Overall, females exhibited a significantly lower mean total 25(OH)D concentration compared to males (65.37 ± 24.91 versus 88.24.04 nmol/L respectively, *p* < 0.001). A significantly greater proportion of female athletes were sampled during the autumn/winter compared to male athletes (*n* = 36 and *n* = 16 respectively, *p* < 0.001). In total, 25% of athletes reported consuming vitamin D supplements in the 6 months prior to sampling. There was no difference in the ratio of athletes reporting/not reporting vitamin D supplement use between sport disciplines, Table 2.

Vitamin D supplementation was a significant positive predictor of vitamin D status (Table 3).

Equatorial travel and sunbed use were reported in 47% and 16% of athletes respectively. A higher ratio of rugby players and cricketers reported equatorial travel compared to boxers and a higher ratio of rugby players and boxers reported sunbed use compared to cricketers (Table 2), but these factors did not predict vitamin D status (Table 3).

## 4. Discussion

The terminology pertaining to deficient/insufficient/sufficient vitamin D status remains a topic of ongoing debate, with no widely accepted guidelines in place for athletes [12]. According to U.S Institute of Medicine guidelines, 14% of athletes in the current study exhibited vitamin D insufficiency/deficiency; a stark difference from 55% when elite Irish athletes were tested in 2010/2011, albeit not in the same cohort [10]. Elite boxers were tested in both studies and, in this group specifically, the prevalence of vitamin D insufficiency/deficiency has decreased from 29% to 10% since 2010/2011. On average, athletes from all sport disciplines were vitamin D sufficient. Although rugby seven players had a lower mean total 25(OH)D concentration than those from other sport disciplines, this is unlikely to be clinically meaningful; certainly with respect to bone health. Furthermore, this difference can likely be attributed to a greater proportion of female athletes being sampled during autumn/winter than male athletes.

Whilst studies around the globe have demonstrated that vitamin D insufficiency/deficiency is a health concern affecting athletes [2]; not all elite cohorts have exhibited clinical deficiency, in support of the current findings [15,16,17,18,19,20,21,22]. Unlike the majority of athletes, those competing at the elite level have access to a support network of dietitians that regularly monitor dietary intake and nutrient status by analysis of food diaries and blood screening. Since the findings of Magee and colleagues [10], total 25(OH)D concentrations have been included as part of routine blood screening for elite athletes in Ireland; thereby enabling targeted supplementation practices. Vitamin D supplement use was confirmed as a positive predictor of vitamin D status in the current study. This finding reinforces the important role that vitamin D supplementation has to play in the optimisation of elite athletes vitamin D status. Elite Irish athletes are routinely screened during the late autumn/early winter (October onward) when ultraviolet B radiation is no longer able to trigger dermal vitamin D synthesis and risk of deficiency increases concomitantly [23]. This marks a change in strategy from pre-2010/2011 when vitamin D screening and supplementation was uncommon. Athletes that exhibit a total 25(OH)D concentration below 125 nmol/L are currently assigned to a supplementation protocol based upon their initial total 25(OH)D concentration. This targeted approach likely decreases the risk of inadvertent vitamin D toxicity (total 25(OH)D concentration ≈ 250 nmol/L) through excessive supplementation when it is not required [24]. Total 25(OH)D is quantified in athlete’s serum using liquid chromatography-tandem mass spectrometry (LC-MS/MS); a method that is widely regarded as the current gold-standard [25]. Alternative analytical methods, such as immunoassay, can overestimate 25(OH)D concentrations leading to misdiagnosis [26].

There is growing evidence that vitamin D_3_ (cholecalciferol) has superior bioavailability compared to vitamin D_2_ (ergocalciferol) as a result of stronger association to vitamin D binding protein [27]. This concept has been corroborated by several randomised controlled trials demonstrating that vitamin D_3_ can be up to 70% more effective than vitamin D_2_ at increasing total 25(OH)D [28,29]. As such, elite Irish athletes are supplemented with vitamin D_3_.

The Irish Sports Council promotes a ‘food-first’ approach with regard to dietary supplements [30]. This is a precautionary stance consistent with other National Governing Body policies worldwide owing to numerous reports of supplement contamination with World Anti-Doping Agency (WADA)-banned substances [31,32,33,34,35]. Although there are a growing number of vitamin D-fortified products available in Ireland [36,37], there are few naturally-occurring dietary sources of vitamin D and intake of such foods is low in the typical Western diet [38,39]. It therefore remains challenging for athletes to attain the current UK reference nutrient intake of 10 µg/day from diet alone [10,13]. In the absence of widespread vitamin D fortification; this conundrum may be best resolved through athlete’s consultation with the team dietitian and provision of batch-tested supplements that are confirmed to be free of contaminants. In the United Kingdom and Ireland, vitamin D_3_ supplements certified as contaminant-free by Informed Sport are currently available in doses ranging from 1000 IU (25 µg) to 5000 IU (125 µg) [40,41,42]. It should be noted however that the tolerable upper intake limit for vitamin D has been set at 4000 IU (100 µg)/day by the European Food Safety Authority [43].

Strengths of this study include the large sample size and recruitment of both male and female athletes from several high-profile sport disciplines. A potential limitation is the self-reported measure of equatorial travel. Although 47% of athletes reported equatorial travel, this was not deemed a significant predictor of vitamin D status. Although this may seem surprising owing to the unequivocal role of UVB exposure in vitamin D synthesis [44]; one should not assume that equatorial travel for training/competition invariably results in an enhanced vitamin D status. It is plausible to suggest that effective application of high sun protection factor cream and indoor training, to avoid excessive heat exposure during peak sunlight hours, may limit vitamin D synthesis despite an equatorial location [45]. The lifestyle questionnaire in the current study had not been previously validated and therefore this could be deemed a potential limitation. Future studies may wish to consider assessing the habitual dietary intake of vitamin D-containing foods by elite athletes, albeit expected to make a negligible contribution to overall vitamin D status [10,13] and using dosimeters to quantify UVB exposure of athletes whilst away on training camps and competition. In summary, vitamin D sufficiency is now commonplace in elite Irish athletes. This finding demonstrates the efficacy of targeted supplementation in elite sport and suggests that blanket-supplementation of elite Irish athletes may therefore not be appropriate.

## Figures and Tables

**Table 1:** Physical and biochemical characteristics of elite athletes presented as mean ± SD.

Measure	Total Samples (*n* = 92)	Rugby (*n* = 43)	Boxing (*n* = 21)	Cricket (*n* = 28)	*p* ^a^
Age, y	25 ± 5	25 ± 4	23 ± 4 ^d^	28 ± 7 ^c^	0.021
Height, cm	175 ± 9	168 ± 6 ^c,d^	179 ± 10 ^b^	182 ± 6 ^b^	0.003
Weight, kg	75.79 ± 14.12	68.05 ± 6.64 ^d^	74.52 ± 16.87 ^d^	88.64 ± 11.16 ^b,c^	0.003
BMI, kg/m^2^	24.55 ± 2.95	23.83 ± 1.67 ^d^	22.96 ± 2.88 ^d^	26.85 ± 3.25 ^b,c^	0.003
25(OH)D_2_, nmol/L	2.02 ± 1.54	2.05 ± 1.36	2.76 ± 1.76 ^d^	1.42 ± 1.43 ^c^	0.006
25(OH)D_3_, nmol/L	74.48 ± 27.54	64.16 ± 24.73 ^d^	81.27 ± 34.46	85.24 ± 20.00 ^c^	0.003
Total 25(OH)D, nmol/L	76.50 ± 27.00	66.20 ± 24.44 ^c,d^	84.03 ± 33.20 ^b^	86.66 ± 19.78 ^b^	0.003
Adjusted calcium, mmol/L	2.30 ± 0.17	2.26 ± 0.13	2.33 ± 0.08	2.32 ± 0.10	0.060
PTH, pg/mL	32.71 ± 11.54	34.69 ± 12.82	31.08 ± 12.18	30.84 ± 8.49	1.000

^a^ Differences between sport disciplines, ANOVA with Bonferroni post-hoc test. *p* value corrected for multiple comparisons; ^b^ Significantly different from rugby, *p* < 0.05; ^**c**^ Significantly different from boxing, *p* < 0.05; ^**d**^ Significantly different from cricket, *p* < 0.05.

**Table 2:** Number of athletes reporting equatorial travel, vitamin D supplementation and sunbed use in the 6 months prior to sampling.

	Total Cohort (*n* = 92)	Rugby (*n* = 43)	Boxing (*n* = 21)	Cricket (*n* = 28)	*p* ^a^
Measure	Yes/No	Yes/No	Yes/No	Yes/No	
Equatorial travel	43:48	24:19 ^b^	2:18	17:11 ^b^	0.006
Vitamin D supplement use	23:68	12:31	6:14	5:23	1.00
Sun bed use	15:76	8:35 ^c^	7:13 ^c^	0:28	0.030

^a^ Chi-square test with Bonferroni post-hoc test. *p* value corrected for multiple comparisons; ^b^ Yes/No responses were significantly different from boxing, *p* < 0.05; ^**c**^ Yes/No responses were significantly different from cricket, *p* < 0.05.

**Table 3:** Logistic regression model predicting likelihood of athletes having a total 25(OH)D concentration above 50 nmol/L.

Predictor	β	S.E	Wald	*p*	Odds Ratio	95.0% C.I. for Odds Ratio	
						Lower	Upper
Vitamin D supplementation	1.46	0.66	4.88	0.027 ^a^	4.31	1.18	15.75
Equatorial travel	0.23	0.65	0.13	0.720	1.26	0.35	4.49
Sunbed use	−1.18	1.12	1.12	0.291	0.29	0.03	2.75

^a^ Statistically significant predictor of a total 25(OH)D concentration above 50 nmol/L, *p* < 0.05.

## Abbreviations

BMI	body mass index
25(OH)D_2_	25-hydroxyvitamin D_2_
25(OH)D_3_	25-hydroxyvitamin D_3_
PTH	parathyroid hormone
